# Long-Term Engraftment and Satellite Cell Expansion from Human PSC Teratoma-Derived Myogenic Progenitors

**DOI:** 10.3390/cells14151150

**Published:** 2025-07-25

**Authors:** Zahra Khosrowpour, Nivedha Ramaswamy, Elise N. Engquist, Berkay Dincer, Alisha M. Shah, Hossam A. N. Soliman, Natalya A. Goloviznina, Peter I. Karachunski, Michael Kyba

**Affiliations:** 1 Lillehei Heart Institute, University of Minnesota, Minneapolis, MN 55455, USA; khosr041@umn.edu (Z.K.); engquist@umn.edu (E.N.E.); bdincer@umn.edu (B.D.); nagyx051@alumni.umn.edu (H.A.N.S.);; 2 Department of Pediatrics, University of Minnesota, Minneapolis, MN 55455, USA; 3 Greg Marzolf Jr. Muscular Dystrophy Center, University of Minnesota, Minneapolis, MN 55455, USA; karac001@umn.edu; 4 Department of Neurology, University of Minnesota, Minneapolis, MN 55455, USA

**Keywords:** skeletal muscle, regeneration, teratoma, transplantation, xenograft, satellite cells, iPS cells, PAX7

## Abstract

Skeletal muscle regeneration requires a reliable source of myogenic progenitor cells capable of forming new fibers and creating a self-renewing satellite cell pool. Human induced pluripotent stem cell (hiPSC)-derived teratomas have emerged as a novel in vivo platform for generating skeletal myogenic progenitors, although in vivo studies to date have provided only an early single-time-point snapshot. In this study, we isolated a specific population of CD82^+^ ERBB3^+^ NGFR^+^ cells from human iPSC-derived teratomas and verified their long-term in vivo regenerative capacity following transplantation into NSG-mdx^4Cv^ mice. Transplanted cells engrafted, expanded, and generated human Dystrophin^+^ muscle fibers that increased in size over time and persisted stably long-term. A dynamic population of PAX7^+^ human satellite cells was established, initially expanding post-transplantation and declining moderately between 4 and 8 months as fibers matured. MyHC isoform analysis revealed a time-based shift from embryonic to neonatal and slow fiber types, indicating a slow progressive maturation of the graft. We further show that these progenitors can be cryopreserved and maintain their engraftment potential. Together, these findings give insight into the evolution of teratoma-derived human myogenic stem cell grafts, and highlight the long-term regenerative potential of teratoma-derived human skeletal myogenic progenitors.

## 1. Introduction

Skeletal muscle, comprising approximately 40% of adult human body weight, consists of both cells providing contractile function, i.e., myofibers, and supportive cells, including satellite cells, which are marked by PAX7 expression [1], reside beneath the basal lamina of muscle fibers [2], play a crucial role in regeneration by responding to injury by repairing and regenerating new muscle fibers [3,4]. Satellite cells also contribute myonuclei to existing fibers during hypertrophic remodeling [5]. Satellite cells can be transplanted into injured muscle, where they will both regenerate fibers and contribute to the satellite cell pool [6,7]. However, the limited number of satellite cells in adult tissue—comprising only 1–2% of the mononuclear cell population—and the fact that muscle tissue is destroyed in their harvest, limits their clinical application for therapeutic purposes.

**Figure 1:**
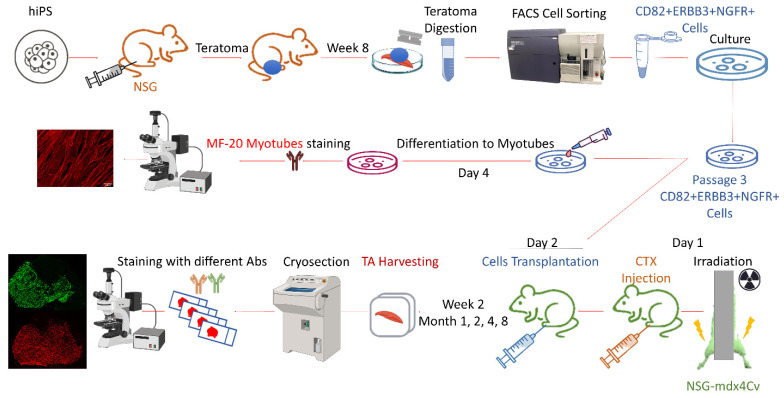
Scheme outlining the key steps of the study. hiPSCs were injected into TA muscles of NSG mice, and teratomas were harvested two months later. Tissue was digested, and skeletal myogenic progenitor cells co-expressing CD82, ERBB3, and NGFR were sorted by FACS, cultured in vitro, tested for differentiation into myotubes, and, in parallel, transplanted into tibialis anterior (TA) muscles of injured NSG-mdx^4Cv^ mice following X-ray and cardiotoxin injection. TA muscle tissue was harvested at various time points, and stained sections were analyzed with different antibodies to assess human cell contribution, muscle regeneration, and fiber type differentiation.

One promising strategy for treating genetic disease of muscle is to replace diseased fibers with healthy ones by transplanting myogenic progenitors derived from human induced pluripotent stem cells (hiPSCs). iPSC can be generated from cells obtained from skin biopsies, i.e., without severely injuring the donor, and can be directed to differentiate into skeletal myogenic progenitors, which will regenerate dystrophic muscle with wild-type fibers and satellite cells [8]. Protocols for in vitro differentiation of hiPSCs into myogenic progenitors tend to be complex, expensive, and subject to variability [8,9,10,11]. We explored a simple in vivo alternative to in vitro differentiation, namely, allowing masses of implanted PSCs to differentiate in immune-deficient animals where they form teratomas, which are tumors that contain cell types from all three germ layers [12]. Mouse PSC-derived teratomas are actually a rich source of skeletal myogenic progenitors [13], and more recently, human PSC-derived teratomas have been shown also to contain skeletal myogenic progenitors that could be isolated by flow cytometry using antibodies to CD82 and ERBB3 [14]. Using a third antigen, NGFR, a very selective subpopulation could be isolated that expanded in vitro and, when transplanted, exhibited a regenerative potential, expressed in terms of sheer mass of new muscle generated, similar to endogenous satellite cells [15]. This exciting new approach has many potential applications, among which are providing a pathway for regenerating damaged muscle tissue and restoring function of diseased muscles, and generating human xenografts to study human disease in vivo in ways that are not feasible or ethical in humans. Both of these applications require not only that human muscle be generated, but that that muscle be populated with human satellite cells, in order to enable long-term maintenance and regeneration after subsequent muscle injury. Previous research has demonstrated that these teratoma-derived progenitors, upon transplantation, can regenerate significant muscle mass; however, other than documenting their presence microscopically, the PAX7+ cell frequency in these grafts is unknown, and the longevity and evolution of these grafts is unknown. In the current study (schematized in Figure 1), we address these critical questions, namely, the long-term engraftment potential of this novel xenograft approach, its evolution in terms of maturation state, and the extent and change over time of the PAX7+ compartment.

## 2. Materials and Methods

### 2.1. Mice

Both male and female NSG and NSG-mdx^4Cv^ mice, aged 3 to 4 months, were utilized as xenograft recipients. Mouse work was conducted under animal protocol 2307-41280A, approved by the Institutional Animal Care and Use Committee of the University of Minnesota.

### 2.2. Human-Induced Pluripotent Stem Cell (hiPSC) Culture

iPSCs were derived from primary cells grown from a skin biopsy sample, obtained under a human subjects protocol (STUDY00000409) approved 19 June 2018 by the University of Minnesota IRB, and reprogrammed using CytoTune™-iPS 2.0 Sendai Reprogramming Kit (A16517, Thermo Fisher Scientific, Waltham, MA, USA), according to the manufacturer’s instructions. Undifferentiated iPSCs were maintained in mTeSR1 medium (STEMCELL Technologies, Vancouver, BC, Canada) on Matrigel-coated flasks (CB40234, Corning, NY, USA). Passaging was performed using Accumax (SCR006, EMD Millipore, Burlington, MA, USA) upon reaching 50% confluency.

### 2.3. Teratoma Formation

Anesthesia was administered to NSG mice via intraperitoneal injection of a ketamine (150 mg/kg, i.p., Akorn #NDC: 59399-114-10, Lake Forest, IL, USA) and xylazine (10 mg/kg, i.p., Akorn #NDC: 59399-111-50) cocktail. One million undifferentiated hiPSCs were prepared in mTeSR1 medium and mixed with Matrigel at a 1:1 ratio (total volume 20 μL). The cell–Matrigel mixture was then injected into the tibialis anterior (TA) muscles using a 28 G Hamilton syringe (cal7636-01, Hamilton Company, Reno, NV, USA).

### 2.4. Teratoma Harvest

Teratomas were harvested 8 weeks after transplantation, minced into pieces smaller than 2 mm, and incubated while shaking in 10 mL of 0.2% collagenase type II (17101-015, Gibco, Grand Island, NY, USA) in DMEM/High Glucose medium (Gibco™ #21063029) at 37 °C. After 40 min, the digestion was stopped by washing with rinsing solution (F-10 +, Ham’s/F-10 medium (MT10070CV, Corning Inc., Corning, NY, USA) supplemented with 10% Horse Serum (SH30074.03, Gibco, Grand Island, NY, USA), 1% 1 M HEPES Buffer Solution (Gibco, 15630-106), and 1% Pen/Strep (Gibco, CX30324)), and digested tissue centrifuged at 1500 rpm for 5 min at 4 °C. Subsequently, the tissue was incubated while shaking in 5 mL of 0.2% Dispase (Gibco, 17105-041) in DMEM/High Glucose medium (Gibco™ #21063029) at 37 °C for an additional 20 min. After vortexing, the samples were drawn and released using an 18-gauge needle four times, then filtered through 40 μm cell strainers and washed with rinsing solution. The cell suspension was centrifuged at 1500 RPM for 5 min, resuspended in FACS buffer (0.2% Fetal bovine serum (FBS) (PS-FB3, PEAK Serum, Wellington, New Zealand) in PBS (MT21040CV, Corning Inc., Corning, NY, USA) for FACS analysis.

### 2.5. Fluorescence-Activated Cell Sorting (FACS)

The dissociated cells were incubated with antibodies APC anti-CD82 (FAB4616A, R&D Systems, Minneapolis, MN, USA; RRID: AB_2076404), PE anti-ERBB3 (304706, BioLegend, San Diego, CA, USA; RRID: AB_2099569), and PECy7 anti-NGFR (Cat# 562122, BD Biosciences, San Jose, CA, USA; RRID: AB_10894762); each at 0.5 uL per 1 million cells) in FACS buffer for one hour on ice. To distinguish live cells, Propidium iodide (PI) (1 μg/mL, Sigma #P4170, Saint Louis, MO, USA) was added to the FACS buffer. Only live cells (PI−) were included in the analysis. FACS analysis and cell sorting were conducted using a BD FACSAriaII (BD Biosciences, San Diego, CA, USA) equipped with FACSDiva software (BD Biosciences). Flow cytometry plots were generated using FlowJo (FLOWJO LLC, Ashland, OR, USA).

### 2.6. Myogenic Cell Culture

CD82+ ERBB3+ NGFR+ (2 × 10^4^) cells sorted from teratomas were plated into each well of a 48-well plate, pre-coated with 0.1% gelatin, and cultured under different experimental conditions. The cells were cultured in SkGM-2 medium (Lonza #CC-3245, Switzerland), which contains epidermal growth factor (EGF), and supplemented with β-nerve growth factor (β-NGF) at 1 ng/mL and basic fibroblast growth factor (bFGF) at 10 ng/µL. Once the cultures reached 80% confluence, cells were passaged by rinsing with PBS and treating them with 0.25% Trypsin-EDTA (Gibco™ #25200056) at 37 °C for 3 min.

### 2.7. Cryopreservation and Thawing of Myogenic Progenitors

P1 cells at 80% confluence were detached by rinsing with PBS and treating with Trypsin-EDTA. After counting, the cells were cryopreserved in a freezing solution consisting of 90% FBS and 10% dimethyl sulfoxide (DMSO) (CMX-30145, Chem-Impex International, Wood Dale, IL, USA). After thawing, the cells were cultured, passaged at 80% confluence, and subsequently used for transplantation at P4.

### 2.8. In Vitro Myotube Differentiation

Cells were cultured in 96-well plates at passage 3. Upon reaching approximately 90% confluency, the proliferation medium (SKGM-2) was replaced with myogenic differentiation medium composed of DMEM/F12 supplemented with 1% Glutamax (SCR006, Life Technologies, Carlsbad, CA, USA), 1% N2 (17502-048, Gibco, Grand Island, NY, USA), 1% ITS-A (51300-044, Gibco, Grand Island, NY, USA), 1% insulin-transferrin-selenium (51300-044, Gibco, Grand Island, NY, USA), 1% penicillin/streptomycin (Life Technologies, #15140-122), and 10 µM SB-431542 (A8249, APExBIO, Houston, TX, USA). After 4 days, cells were fixed and immunostained for myosin heavy chain (clone MF20, DSHB, Iowa City, IA, USA), MyoD (554130, BD Pharmingen™, San Jose, CA, USA), Myogenin (ab124800, Abcam, Cambridge, MA, USA), and PAX7 (AB_528428, DSHB, Iowa City, IA, USA). Additionally, cells at the same passage used for transplantation were stained for PAX7 during the proliferative phase (SKGM-2 medium) at confluency, without switching to differentiation medium. For each marker, three wells were stained, and five distinct fields were imaged per well. Cell quantification was performed using ImageJ2 (v3.2.0, NIH, Bethesda, MD, USA).

### 2.9. Muscle Transplantation

NSG-mdx^4Cv^ mice were anesthetized with ketamine (150 mg/kg, i.p., Akorn #NDC:59399-114-10, Lake Forest, IL, USA) and xylazine (10 mg/kg, i.p., Akorn #NDC:59399-111-50). Both hindlimbs were then exposed to 600 cGy X-ray irradiation. To induce muscle injury, 15 μL of cardiotoxin (10 μM, Latoxan #L8102, France) was injected into both the left and right TA muscles on the same day. The next day, 200,000 cells in SkGM-2 medium (Lonza #CC-3245, Basel, Switzerland) with Y27632 (10 nM, APExBio #A3008, Houston, TX, USA) were transplanted into the TA muscles using a 28 G Hamilton syringe (cal7636-01, Hamilton Company, Reno, NV, USA). Mice were randomly assigned to experimental or control groups, with controls being injected with PBS. Engraftment was evaluated at multiple time points: 2 weeks, and 1, 2, 4, and 8 months post-transplantation, with three samples analyzed at each time point.

### 2.10. Immunostaining on Cultured Cells

Cell cultures were fixed with 4% paraformaldehyde (PFA) (sc-253236B, Santa Cruz Biotechnology, Dallas, TX, USA) at room temperature for 20 min. To permeabilize the cells, 0.3% Triton X-100 (22140, Electron Microscopy Sciences, Hatfield, PA, USA) was applied for 30 min. The cells were then blocked with 3% bovine serum albumin (BSA) (A-420-500, Gold Biotechnology, St. Louis, MO, USA) for one hour. After blocking, specific primary antibodies diluted in 3% BSA were applied for overnight staining at 4 °C. This was followed by incubation with secondary antibodies (also diluted in 3% BSA) for one hour at room temperature and counterstained with DAPI for 10 min. The primary antibodies included rabbit anti-MYOD1 (sc-304, Santa Cruz Biotechnology, Dallas, TX, USA; used at 1:500 dilution), rabbit anti-myogenin (ab124800, Abcam, Cambridge, MA, USA; used at 1:500 dilution), and mouse anti-MHC (clone MF-20, DSHB, Iowa City, IA, USA; used at 1:20 dilution). The secondary antibodies were goat anti-mouse IgG Alexa Fluor 555 and goat anti-rabbit Alexa Fluor 647 (Life Technologies, Carlsbad, CA, USA). Images were captured using a Zeiss AxioObserver Z1 inverted microscope equipped with an AxioCam MR3 camera (Carl Zeiss Microscopy GmbH, Jena, Germany), and ZEN software (version 3.2) was used for image acquisition and processing.

### 2.11. Immunostaining on Muscle Sections

TA muscles were harvested for analysis at different time points post-transplantation. The harvested TA muscles were embedded in optimal cutting temperature (OCT) solution (23-730-625, Fisher Scientific, Waltham, MA, USA), snap-frozen in liquid nitrogen-chilled 2-methylbutane (O35514, Cole-Parmer, Vernon Hills, IL, USA), and stored at −80 °C. Muscle sections were cut at 10 μm thickness using an Epredia™ CryoStar™ NX50 cryostat (Epredia, Kalamazoo, MI, USA) and collected in serial sections. The sections were fixed with 4% PFA, rehydrated with PBS, permeabilized with 0.3% Triton X-100 for 30 min, and blocked with 3% BSA for 1 h at room temperature. Primary antibodies diluted in 3% BSA were applied to the sections and incubated overnight at 4 °C, followed by secondary antibody incubation for 1 h at room temperature. DAPI was used to stain the nuclei. The slides were mounted using Immu-Mount (9990402, Thermo Scientific, Waltham, MA, USA), and images were captured in tile mode using ZEN software (version 3.2, Carl Zeiss Microscopy GmbH, Jena, Germany) on a Zeiss AxioObserver Z1 inverted microscope (Carl Zeiss Microscopy GmbH, Jena, Germany), and the measurements were performed using ImageJ software (ImageJ2 v3.2.0, NIH, Bethesda, MD, USA). The individual doing the staining was blinded to the conditions represented by each sample.


**Primary antibodies:**
Rabbit anti-DYSTROPHIN (1:250, Abcam, #ab15277).Mouse anti-hLamin A/C (1:100, ThermoFisher Scientific, #mab636; AB_325377).Rabbit anti-MyoD Antibody (C-20) (1:500, Santa Cruz Biotechnology, #sc-304).Mouse anti-Dystrophin (MANDYS106, 2C6) (1:100, DSHB, #AB_2753249).Rabbit anti-hLamin A/C (1:500, Abcam, #ab108595).Rabbit anti-laminin (1:500, Sigma, #L9393).Rat anti-Laminin α-2 (4H8-2) (1:200, Santa Cruz Biotechnology, #sc-59854).Mouse anti-PAX7 (1:10, DSHB, #AB_528428).Mouse anti-neonatal MHC (N3.36) (1:20, (MIgM) DSHB, #AB_528380).Mouse anti-embryonic MHC (F1.652) (1:20, (MIgG1) DSHB, #AB_528358).Mouse anti-MHC-I slow (BA-F8) (1:20, (MIgG2b) DSHB, #AB_10572253).Mouse anti-MHC-IIA (SC-71) (1:20, (MIgG1) DSHB, #AB_2147165).Mouse anti-MHC-IIB (BF-F3) (1:20, (MIgM) DSHB, #AB_2147165).
**Secondary antibodies:**
Goat anti-rabbit Alexa Fluor 488 (111-545-144, Jackson ImmunoResearch Laboratories, West Grove, PA, USA).Goat anti-mouse Alexa Fluor 555 (A21422, Life Technologies, Carlsbad, CA, USA).Goat anti-rat Alexa Fluor 647 (A21247, Invitrogen, Carlsbad, CA, USA).Goat anti-mouse IgM Alexa Fluor 555 conjugate (A21426, Thermo Fisher Scientific, Waltham, MA, USA).Goat anti-mouse IgG1 Alexa Fluor 488 (A21121, Thermo Fisher Scientific, Waltham, MA, USA).Goat anti-mouse IgG2b Alexa Fluor 350 (A21140, Thermo Fisher Scientific, Waltham, MA, USA).

### 2.12. Myofiber Engraftment Area Measurement

The engraftment areas, size of myofibers, quantities of hLaminA/C+ (human) and PAX7+ cells, and expression of myosin heavy chain (MYHC) isoforms across different groups were measured in each section using ImageJ software. Engraftment areas, relative to the total cross-sectional area of the TA section, were visualized using human-specific Dystrophin (MANDYS) and hLamin A/C staining.

### 2.13. Statistical Analysis

Data are presented as mean ± SEM. All statistical analyses were performed using GraphPad Prism v8.02 (GraphPad Software, La Jolla, CA, USA). Significance was calculated using one-way ANOVA with Tukey’s post hoc test for comparison among the groups. Differences are considered to be statistically significant at the *p* < 0.05 level: * *p* < 0.03, ** *p* < 0.002, *** *p* < 0.0002, **** *p* < 0.0001.

### 2.14. Bioinformatics

Sequencing was performed by Genewiz/Azenta (https://www.genewiz.com) (accessed on 7 July 2025). Raw reads were trimmed with Trim Galore (v0.6.10) (http://www.bioinformatics.babraham.ac.uk/projects/trim_galore/)(accessed on 7 July 2025), using Cutadapt (v5.0) [16] to remove Illumina Sequencing adapters from the 3′ end. Quality control was performed using FastQC (v12.1) (http://www.bioinformatics.babraham.ac.uk/projects/fastqc/)(accessed on 7 July 2025), and 15 bases were trimmed from the 5′ end of reads due to biased per base sequence content. Human genome sequence GRCh38 (v113) was downloaded from Ensembl, and reads were mapped to the human transcriptome using Salmon (v1.10.3) [17], employing the GC bias correction flag. Transcript-level reads were aggregated to the gene-level in R using the tximeta package (v1.22.1 [18] and normalized using DESeq2 (v1.44.0) [19]. Data is available in the GEO database (https://www.ncbi.nlm.nih.gov/geo/)(accessed on 7 July 2025) under accession number GSE301588.

## 3. Results

### 3.1. Teratoma Formation and Harvest

**Figure 2:**
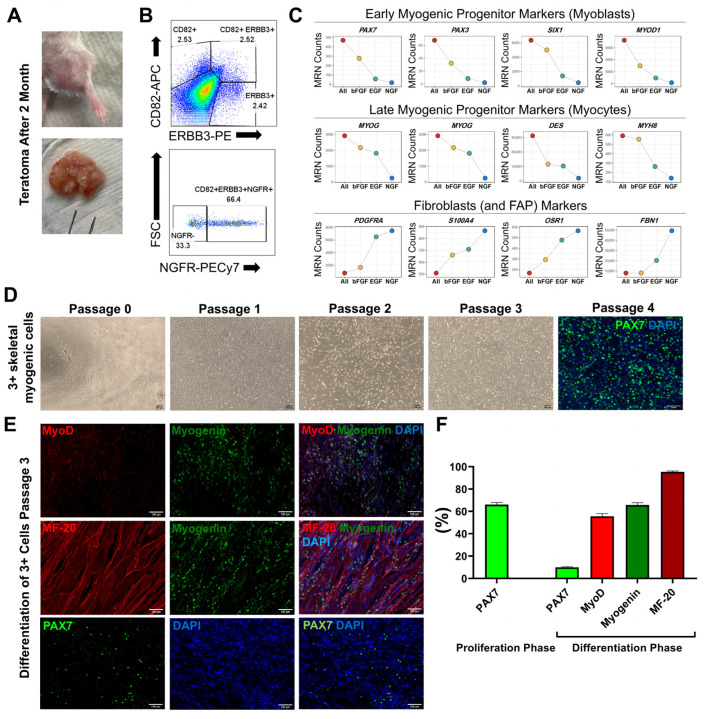
(**A**) Morphology of teratomas formed in TA muscles of NSG mice two months after injection of hiPSCs. (**B**) Isolation of skeletal myogenic progenitor cells co-expressing CD82, ERBB3, and NGFR by FACS from TA teratomas. (**C**) Expression levels of various myogenic and fibroblastic markers under different growth factor conditions, as determined by RNA sequencing, presenting mean ratio normalized counts. (**D**) Morphology of isolated skeletal myogenic cells cultured at different passages. PAX7 staining of cells in the proliferative phase at confluency, from the same passage used for transplantation. (**E**) MyoD, Myogenin, MF-20, and PAX7 staining of teratoma-derived skeletal myogenic cells in differentiation medium at passage 3, highlighting their capacity to form myotubes. (**F**) Percentage of cells positive for PAX7 in proliferative and differentiation media, and for MyoD, Myogenin, and MF-20 in differentiation medium. Data were obtained from five fields per sample across three replicates.

We generated the human iPS cell line 19-009 from skin fibroblasts of a normal human donor and used these cells to generate primary teratomas in NSG mice by transplanting 1 M cells with Matrigel into tibialis anterior (TA) muscles. Eight weeks after transplantation, teratomas were visually distinct and measured approximately 2 cm^2^ in size (Figure 2A).

### 3.2. Isolation of CD82+ ERBB3+ NGFR+ Skeletal Myogenic Cells from Teratomas

Teratomas were enzymatically digested and mechanically dissociated to produce a single-cell suspension, stained for CD82, ERBB3, and NGFR, and the triple-positive population, representing approximately 2% of total teratoma-derived cells, isolated by fluorescence-activated cell sorting (FACS) (Figure 2B).

### 3.3. In Vitro Expansion and Differentiation of Teratoma-Derived Myogenic Progenitors

The original expansion method [15] used SkGM-2 medium, which contains EGF; however, as cells were isolated based on NGF expression and since bFGF is a more commonly used growth factor for myogenic progenitors [20], we tested the effect of culturing alternatively in NGF or bFGF, or in the combination of all 3 growth factors. Cells grew best, differentiated best, and expressed highest levels of myogenic markers and lowest levels of fibroblastic markers when all 3 growth factors were present (Figure 2C); therefore, we used this growth factor combination for expansion in all subsequent work.

Triple-positive skeletal myogenic cells demonstrated robust expansion in vitro, achieving confluence within one week at passage zero and subsequently within four days per passage when cultured in SkGM-2 supplemented with NGF and bFGF (Figure 2D). The functional myogenic potential of these cells was assessed through in vitro differentiation, during which they transitioned from a stellate morphology to elongated, multinucleated myotubes within four days following the switch from proliferation medium (SKGM-2) to myotube differentiation medium (Figure 2E). Immunostaining confirmed the expression of sarcomeric myosin heavy chain (MHC), a key marker of skeletal myotube differentiation (Figure 2E). Approximately 95% of the differentiated cells were positive for MHC, indicating a high degree of differentiation efficiency. We further evaluated the expression of PAX7 in both proliferation and differentiation media, while MyoD and Myogenin were assessed under differentiation conditions. A notable decrease in PAX7-positive cells was observed following differentiation into myotubes. In contrast, 65% of the cells were positive for Myogenin and 55% were positive for MyoD, indicating activation of the myogenic differentiation program (Figure 2F).

### 3.4. Engraftment and Long-Term Maintenance of Human Xenografts

**Figure 3:**
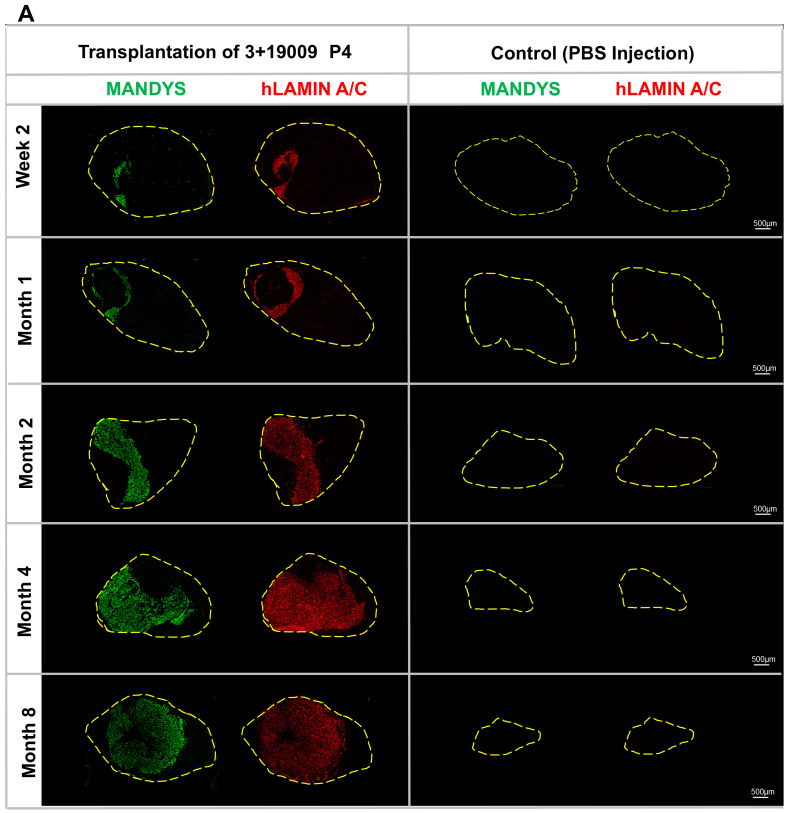

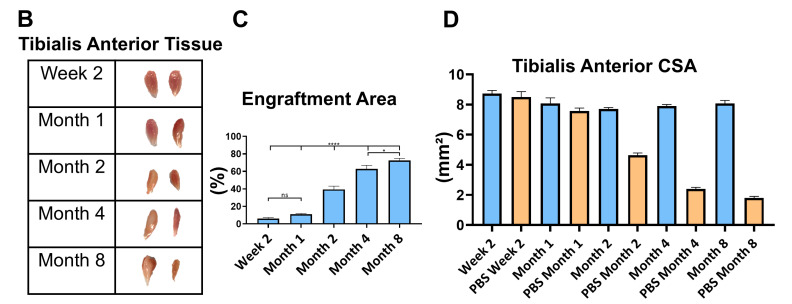
(**A**) Engraftment and muscle size over time, showing both human Dystrophin (MANDYS), which marks fibers, and human-specific Lamin A/C, which marks all human nuclei. Representative images were captured at various time points to assess engraftment in TA muscle sections. The engraftment area was measured relative to the total tissue area in each section. The engrafted area increased progressively over time, reflecting enhanced tissue integration. In contrast, the control group, irradiated and injected with PBS, exhibited a consistent decrease in muscle area, indicating atrophy. The transplanted group, however, maintained stable muscle size, demonstrating the ability of the transplanted cells to preserve muscle integrity and prevent degeneration throughout the study period. Scale bar = 500 µm. (**B**) Morphological changes in the TA muscle over time. (**C**) The percentage of the engrafted area relative to the total TA size at each time point (*p* < 0.0001). (**D**) Quantification of the total TA CSA over time (*p* < 0.0001).

We transplanted 200,000 cells at passage 4 into cardiotoxin (CTX)-injured TA muscles of irradiated NSG-mdx^4Cv^ mice, which lack both functional lymphocytes, allowing tolerance of xenografts, and Dystrophin, allowing unequivocal identification of human myofibers [7]. In order to understand the dynamics of the graft over time and to determine whether it was maintained, engraftment was assessed from two weeks to eight months post-transplantation (Figure 3). Immunostaining of muscle sections with the human-specific nuclear marker hLaminA/C revealed that the transplanted cells engrafted and differentiated, and that the engrafted area expanded over time, being maintained long-term. Staining with the human-specific Dystrophin antibody, MANDYS, showed that the engrafted cells generated human myofibers.

### 3.5. Engraftment Area

To quantify the extent of engraftment at various time points, images were captured from sections of the middle region of each TA muscle. The cross-sectional area (CSA) of the engrafted domain, identified by human-specific MANDYS (green) and human Lamin A/C (red) staining in Figure 3A. The percentage of the engrafted area was measured using ImageJ and compared to the total TA CSA (Figure 3C). For each time point, three TA muscles from three different mice were analyzed.

The transplanted area was approximately 6% of CSA at week 2, increasing to 11% by month 1. By month 2, it expanded to about 39%, reaching 63% at month 4, and ultimately growing to 72% by month 8 (Figure 3C). The total TA CSA was measured using ImageJ and compared across different time points (Figure 3D). The control (PBS-injected) TA muscle displayed progressive atrophy over time. This consistent reduction highlights the ongoing degeneration and loss of muscle tissue in a limb in which endogenous satellite cells have been disabled by irradiation in the absence of therapeutic intervention.

In contrast, the transplanted group displayed remarkable stability in muscle size over the same period. These consistent measurements indicate that the transplanted cells effectively preserved the overall muscle mass, preventing the atrophy observed in the control group. This suggests that the transplanted cells contributed significantly to preventing the atrophy that occurs with time after injury and irradiation.

### 3.6. Engrafted Human Myofibers Increase in Size over Time

**Figure 4:**
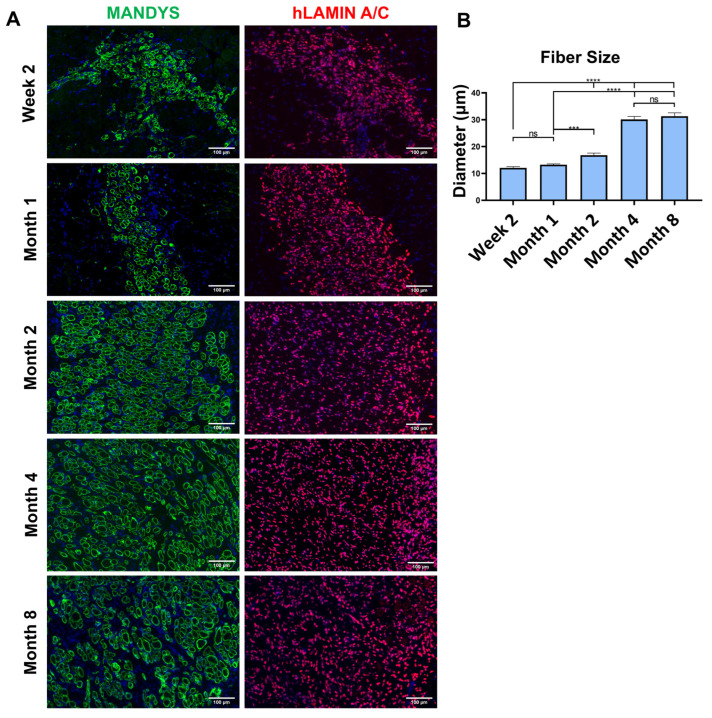
(**A**) Morphology of muscle fibers in transplanted muscles over time. Fiber diameter progressively increased from week 2 through month 4, stabilizing by month 8. These results indicate significant growth up to month 4, followed by a plateau, suggesting sustained muscle regeneration and maturation driven by the transplanted cells. Scale bar = 100 µm. (**B**) Average size (diameter) of engrafted fibers at various time points (*p* < 0.0001).

The size of muscle fibers was measured in 500 fibers from three different fields in each of three samples per group (Figure 4A). The average fiber diameter progressively increased from about 12 µm at week 2 to about 30 µm at month 4, remaining stable to month 8 (Figure 4B). The progressive increase in size suggests that the transplanted cells contribute to sustained muscle regeneration and maturation over time.

### 3.7. Myogenic Differentiation in Transplanted Cells

To further assess the differentiation of the engrafted cells, muscle sections were analyzed via immunostaining for key myogenic markers, including PAX7 for muscle satellite cells and various myosin heavy chain (MyHC) isoforms.

### 3.8. Satellite Cells (PAX7-Positive Cells) and hLamin A/C Expression

**Figure 5:**
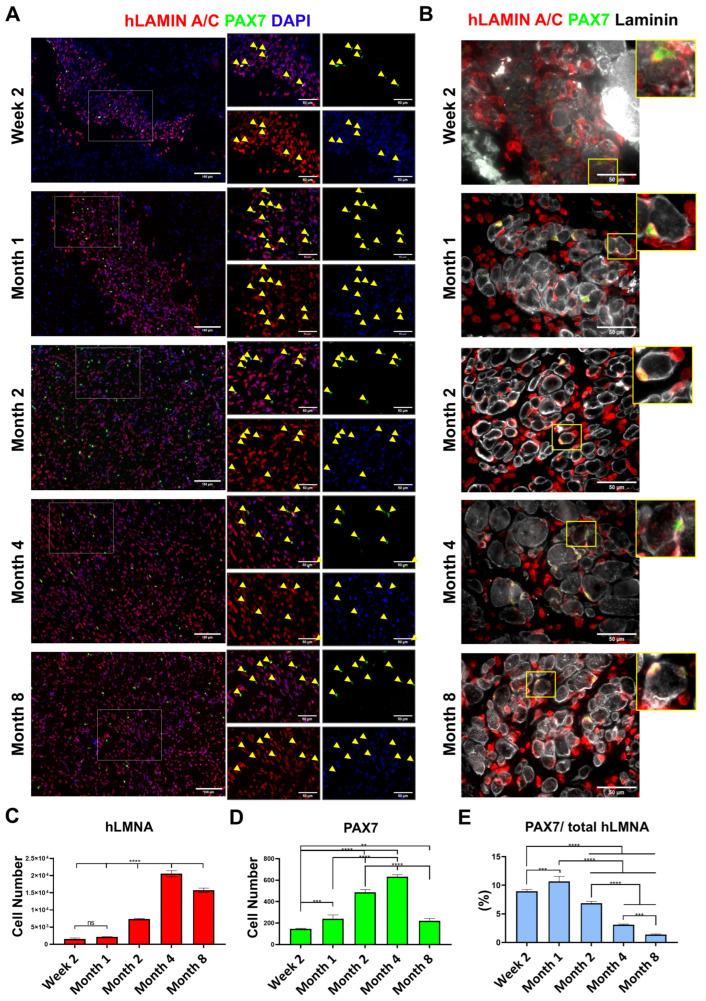
(**A**) PAX7-positive satellite cells in transplanted TA muscles are shown at various time points. Double staining with hLamin A/C and DAPI confirms their human origin and nuclear localization. Yellow arrowheads denote cells positive for PAX7, hLamin A/C, and DAPI staining. Scale bar = 100 µm. (**B**) Double staining with human Lamin A/C, PAX7, and laminin revealed PAX7-positive cells located beneath the basal lamina of newly formed muscle fibers at all time points. However, due to the small size of the fibers at week 2, this localization is less obvious. Scale bar = 50 µm. (**C**) Number of human Lamin A/C-positive cells (hLMNA) at different time points (*p* < 0.0001). (**D**) The number of human PAX7-positive cells per section was quantified over time. All PAX7-positive cells were confirmed to be double-positive for human Lamin A/C at the time of counting (*p* < 0.0001). (**E**) Percentage of human PAX7-positive cells relative to total hLamin A/C-positive cells at various time points (*p* < 0.0001).

Representative images of each TA muscle were captured at multiple time points, with triplicate samples analyzed for each group. PAX7-positive cells were quantified in each section, and their human origin was confirmed by co-expression with hLamin A/C (Figure 5A), validating that the observed PAX7 expression was derived from the transplanted human cells. Notably, no expression of PAX7 or MyHC was detected in the control group, which was injected with PBS alone, underscoring the essential role of transplanted cells in muscle regeneration.

The average number of hLamin A/C-positive cells, representing the total population of transplanted human cells, exhibited a progressive increase over time from about 1.5K cells at week 2 to a peak of just over 20K cells on month 4, reflecting substantial cell expansion and combination into the host tissue (Figure 5C). By month 8, the count slightly decreased, suggesting a stabilization phase or a modest reduction due to tissue remodeling or cell turnover. These data highlight the strong engraftment and proliferative capacity of the transplanted cells during the early and mid-phases of regeneration, followed by a slight decline, in which excess mononuclear cells are depleted, as the graft matures. After transplantation, the number of PAX7-positive cells gradually increased, reaching a peak around month 4, and then declined by month 8 (Figure 5D).

To understand the nature of these engrafted cells as the graft matured, the percentage of PAX7-positive cells relative to the total hLamin A/C-positive cells was calculated. At week 2, PAX7-positive cells comprised approximately 9% of the transplanted cell population, which increased modestly by month 1 (Figure 5E). Subsequently, this proportion began to decline, decreasing by month 2, reaching 3.1% by month 4, and eventually dropping to 1.4% by month 8. For the quantification of PAX7-positive cells, double staining was performed using human Lamin A/C, PAX7, and laminin. PAX7-positive cells were confirmed to be of human origin through co-localization with human Lamin A/C, and their location beneath the basal lamina of newly formed muscle fibers, revealed by laminin staining, strongly supports their identity as satellite cells (Figure 5B).

These results reveal a dynamic pattern of satellite cell activation and differentiation over time. The early increase in both the number and percentage of PAX7-positive cells likely reflects their activation and proliferation in response to the injury microenvironment. The subsequent decline suggests that many of these cells transitioned from a satellite cell-like state to differentiated myogenic lineages, contributing to the formation and maturation of muscle fibers. By month 8, the reduced proportion of PAX7-positive cells indicates that most engrafted cells had either differentiated into mature muscle fibers or had ceased symmetric self-renewal divisions. This pattern highlights the capacity of transplanted cells to not only reload the satellite cell pool but also drive the regeneration of functional muscle fibers, while maintaining a limited reserve of undifferentiated progenitors for potential long-term contributions to muscle repair.

### 3.9. Expression of Myosin Heavy Chain (MyHC) Isoforms

**Figure 6:**
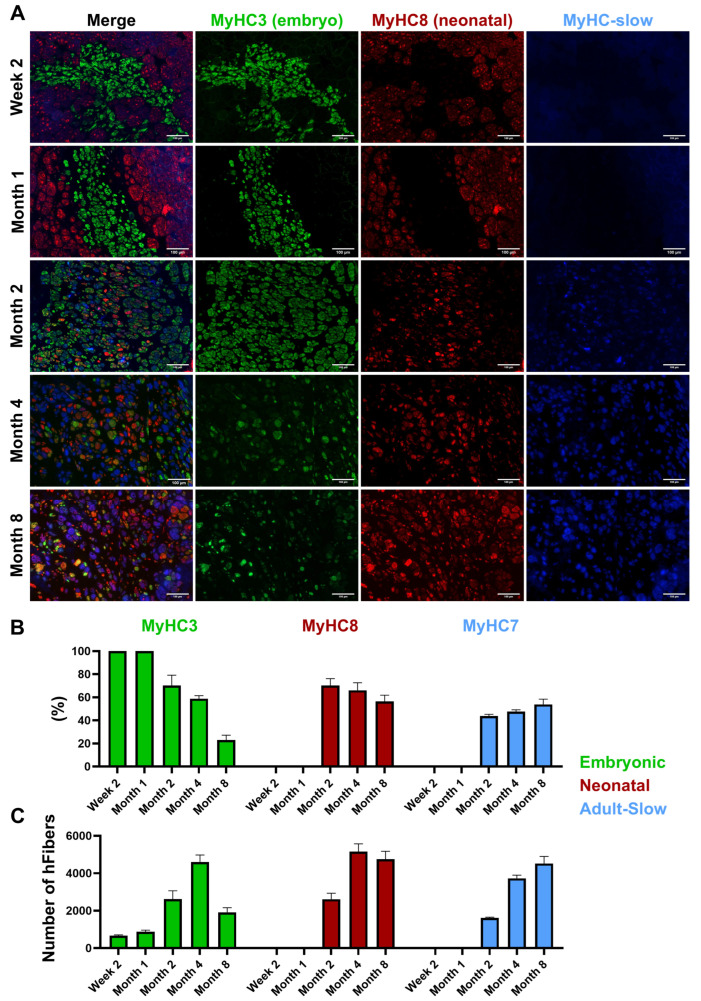
(**A**) MyHC isoform expression over time in transplanted fibers. Scale bar = 100 µm. (**B**) Percentage of newly formed fibers of each fiber type across time points (*p* < 0.0001). (**C**) Total number of newly formed fibers of each fiber type across time points (*p* < 0.0001). At two weeks post-transplantation, all fibers exclusively expressed embryonic MyHC3. By one month, MyHC3 remained predominant. At two months, a subset began expressing neonatal MyHC8 and MyHC-slow, indicating partial maturation. By four months, most fibers expressed neonatal MyHC8 and MyHC-slow, with reduced embryonic MyHC3. At eight months, neonatal MyHC8 and MyHC-slow dominated, with minimal embryonic MyHC3, reflecting sustained maturation.

At two weeks post-transplantation, all muscle fibers derived from the transplanted cells exclusively expressed embryonic myosin heavy chain (MyHC3), with no detectable presence of other MyHC isoforms, indicating an early-stage muscle phenotype (Figure 6A). By one month, the expression pattern remained largely unchanged, with the fibers predominantly expressing MyHC3 (Figure 6B). This consistency suggested the persistence of an embryonic-like state during this period. At two months, a notable shift began to emerge as a subset of fibers started expressing neonatal myosin heavy chain (MyHC8) and MyHC-slow isoforms. Despite the gradual progression of the engrafted cells toward a more mature muscle phenotype, the majority of fibers continued to express MyHC3, highlighting the predominance of embryonic-like character at this stage. By four months post-transplantation, the engraftment area showed significant expansion, accompanied by a substantial change in the MyHC expression profile. A greater proportion of fibers now expressed neonatal MyHC8 and MyHC-slow isoforms, while the number of fibers positive for embryonic MyHC3 noticeably decreased (Figure 6C). This shift suggested an advanced stage of differentiation, with most fibers adopting a neonatal or slow MyHC profile, reflecting further maturation of the transplanted muscle fibers. At eight months, the transplanted cells demonstrated stable expression of neonatal MyHC8 and MyHC-slow isoforms, with only minimal residual expression of embryonic MyHC3. This long-term pattern highlighted the sustained maturation and stabilization of the engrafted fibers, showing that the transplanted cells had largely transitioned to a more mature muscle phenotype over time.

### 3.10. Teratoma-Derived Myogenic Progenitors Retain Their Engraftment Capacity After Freeze/Thaw

**Figure 7:**
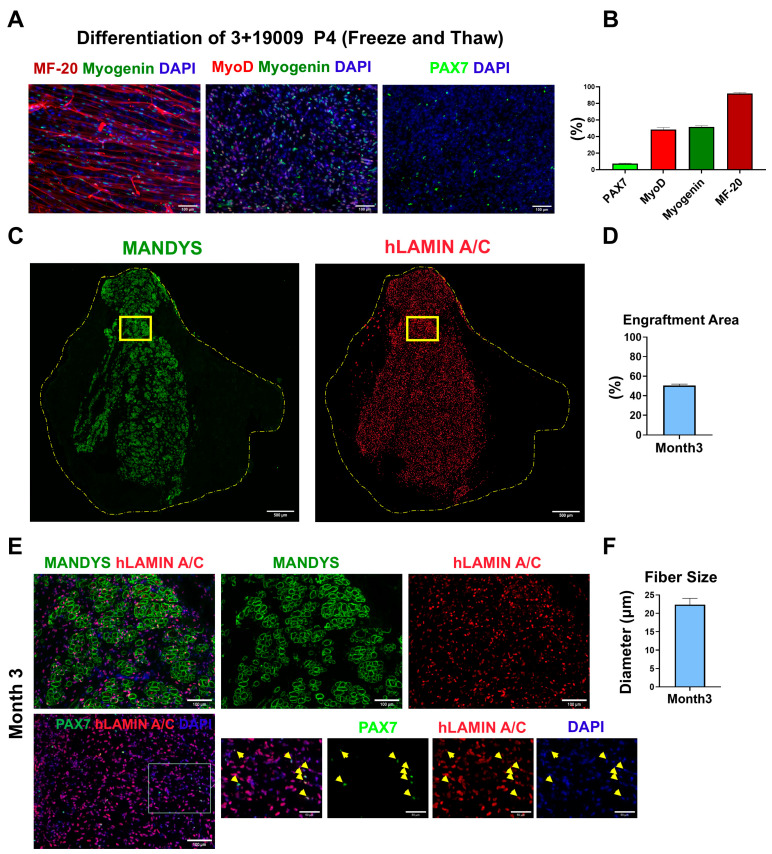

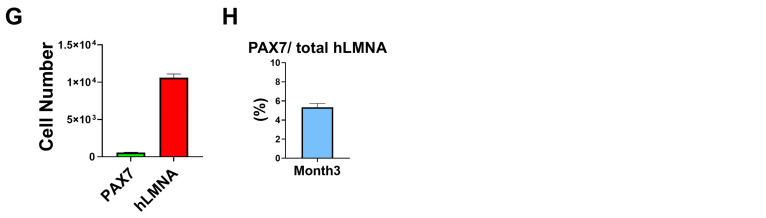
(**A**) MyoD, Myogenin, MF-20, and PAX7 staining of frozen and later thawed teratoma-derived skeletal myogenic cells in differentiation medium at passage 4. Scale bar = 100 µm. (**B**) Percentage of cells positive for PAX7, MyoD, Myogenin, and MF-20 in differentiation medium. Data were obtained from five fields per sample across three replicates. (**C**) Engraftment of frozen and later thawed teratoma-derived skeletal myogenic cells analyzed after three months, showing both human Dystrophin (MANDYS), which marks fibers, and human-specific Lamin A/C, which marks all human nuclei. Representative images were captured to assess engraftment in TA muscle sections. Scale bar = 500 µm. (**D**) The percentage of the engrafted area relative to the total TA size. (**E**) hDystrophin+ fibers and PAX7+ cells in the engrafted area, the human origin double-confirmed by co-staining with hLamin A/C. Yellow arrowheads denote cells positive for PAX7, hLamin A/C, and DAPI staining. Scale bar = 100 µm. (**F**) Average size (diameter) of engrafted fibers. (**G**) Number of human PAX7, hLamin A/C-positive cells (hLMNA). (**H**) Percentage of human PAX7-positive cells relative to total hLamin A/C-positive cells.

Because generation of teratomas is time-consuming, we tested whether a batch of teratoma-derived cells could be cryo-preserved, expanded at a later date, and retain engraftment capacity. We froze triple-positive cells at passage 1 and thawed them one month later. At passage 4, their in vitro myogenic potential was evaluated, and as shown in Figure 7A, these cells successfully differentiated into myotubes, with most nuclei positive for Myogenin. The percentage of cells positive for PAX7, MyoD, Myogenin, and MF-20 in these differentiation cultures is presented in Figure 7B. We also transplanted undifferentiated cells at passage 4, analyzing 3 months post-transplant, and found that they could engraft and form muscle fibers in a manner similar to that seen with fresh cells (Figure 7C). We also confirmed that grafts contained PAX7+ human cells in the engrafted area (Figure 7E). These data suggest that the freeze–thaw process did not compromise the in vivo regenerative potential of the cells.

## 4. Discussion

Induced pluripotent stem cells (iPSCs) present a promising strategy for muscle regeneration by providing a renewable source of myogenic progenitor cells, addressing the deficiency of PAX7+ satellite cells, which are necessary for muscle repair [8,21,22]. While most approaches used are in vitro differentiation methods, the studies described here show that human iPS-derived teratomas provide a source of cells that can be transplanted and will differentiate into muscle fibers that remain for at least 8 months in vivo. These findings support previous research, confirming the value of human PSC-derived teratomas as a reliable source of muscle progenitors [15]. Because previous studies looked at relatively short-term engraftment, here we assessed the long-term stability of these progenitor cells after transplantation, the effects of cryopreservation on their myogenic potential, the presence of PAX7+ cells at different time points, MyHC isoforms, and the evolution of the grafts over time, factors that had not been evaluated previously.

In vitro differentiation assays revealed strong myogenic potential, with the cells forming multinucleated myotubes and expressing key myogenic markers such as MyoD, Myogenin, and MHC. Importantly, these cells retained their myogenic potential after cryopreservation, as evidenced by their ability to differentiate into myotubes in vitro and to promote muscle regeneration in vivo post-thaw. This confirms their viability for long-term storage. Long-term engraftment and survival of transplanted cells are essential for the success of cell-based therapies for skeletal muscle disorders. Effective transplantation requires not only the integration of cells into host muscle but also the establishment of a functional stem cell pool for continuous regeneration [23,24].

Our in vivo transplantation experiments confirmed the regenerative potential of these progenitor cells. Over a four-month period, the engrafted area expanded, and from there remained stable through month 8, indicating successful integration of the transplanted cells into host tissue. Studies show that not only is it important for transplanted muscle progenitor cells to contribute to new muscle fiber formation, but also to their long-term retention by contributing satellite cells, which increases their persistence and functional efficiency over time [25]. In our study, the presence of PAX7+ cells revealed that the transplanted cells not only differentiated into muscle fibers but also expanded as a muscle stem cell population, which besides being important for muscle regeneration [26] is likely contributing to growth in fiber size over time. The dynamic pattern of PAX7 expression, initially increasing and then declining as differentiation progressed, demonstrated that the graft initially drove expansion of mononuclear progenitor cells to an elevated level that was sustained, consistent with regenerative mechanisms seen in muscle injury models [27].

In mdx mice, muscle fiber size exhibits significant variability due to the ongoing cycles of degeneration and regeneration [28]. One study observed that the myofiber diameter begins at approximately 30 µm in 2-week-old mice and progressively increases with age [29]. Another experiment found that in 3-month-old mdx mice, approximately 25% of muscle fibers measured 50–60 µm in diameter, while 10% ranged from 30 to 40 µm, with the remaining fibers exhibiting a range of different sizes, highlighting the significant variation in fiber size within these mice [30]. This heterogeneity reflects the dynamic process of muscle regeneration in mdx mice, where fibers at different stages of maturation coexist due to the continuous cycles of muscle damage and repair resulting from the absence of dystrophin [28,31]. In our study, muscle fiber size increased over time, plateauing at months 4 and 8, suggesting that transplanted cells contributed to sustained muscle regeneration. However, the newly formed fibers were smaller than mature fibers. The transition from a stellate morphology to multinucleated myotubes in vitro and the subsequent increase in fiber size in vivo highlights the differentiation potential of these progenitor cells. Additionally, shifts in MyHC isoform expression confirmed the maturation of transplanted muscle fibers, transitioning from an embryonic phenotype (MyHC3) to a more mature neonatal or slow muscle fiber profile (MyHC8 and MyHC-slow). Delayed maturation is suggested by the persistence of embryonic and neonatal MyHC isoforms in regenerating fibers. This prolonged immature state may delay full fiber hypertrophy, as regenerating fibers must repeatedly undergo repair before reaching full maturation and growth.

Even though the results are encouraging, several aspects need further investigation. In this experiment, we included NGF and bFGF in the culture medium due to their established roles in enhancing cell proliferation and differentiation [20,32]. However, we did not include a control group without these growth factors, making it difficult to completely evaluate their specific effects. Future studies should concentrate on evaluating the impact of different growth factors on these skeletal myogenic progenitor cells, both in vitro and in vivo, to better understand how these factors influence the cells’ regenerative potential. In addition, while our objective here was to develop an improved xenograft assay replete with PAX7+ cells that would better reflect human muscle tissue in vivo, additional work would need to be undertaken to address possible therapeutic potential, which is beyond the scope of the current study. Although the method described here uses wild-type karyotypically normal iPSC-derived teratomas, which are not intrinsically carcinogenic as they lack the genetic and epigenetic changes associated with pathological human primary teratomas [33], much work would need to be carried out to improve the overall characteristics of engraftment including fiber maturation, before the method could be considered for therapeutic transplantation.

In conclusion, this study provides compelling evidence that skeletal myogenic progenitor cells derived from human iPSC-based teratomas exhibit strong in vitro differentiation potential and in vivo regenerative capacity, with stable muscle fiber regeneration over time. This approach thus presents a promising source of myogenic cells, valuable for modeling human muscle in vivo. Further exploration of their therapeutic potential could also open up new possibilities for effective cell-based therapies for muscle diseases and injuries.

## Data Availability

Data associated with this study will be made available upon reasonable request to Michael Kyba.
